# Determining RE-AIM indicators for evaluating the Estratégia Amamenta e Alimenta Brasil (EAAB – Brazilian Breastfeeding and Complementary Feeding Strategy)

**DOI:** 10.11606/s1518-8787.2024058005875

**Published:** 2024-09-16

**Authors:** Daiane Sousa Melo, Sonia Isoyama Venancio, Gabriela Buccini

**Affiliations:** I Universidade de São Paulo Faculdade de Saúde Pública Departamento de Nutrição São Paulo SP Brasil Universidade de São Paulo. Faculdade de Saúde Pública. Departamento de Nutrição. São Paulo, SP, Brasil; II Ministério da Saúde Coordenação de Atenção à Saúde da Criança e do Adolescente Brasília DF Brasil Ministério da Saúde. Coordenação de Atenção à Saúde da Criança e do Adolescente. Brasília, DF, Brasil; III University of Nevada Las Vegas School of Public Health Department of Social and Behavioral Health Las Vegas USA University of Nevada Las Vegas. School of Public Health. Department of Social and Behavioral Health. Las Vegas, USA

**Keywords:** Breast Feeding, Infant Nutritional Physiological Phenomena, Primary Health Care, Implementation Science

## Abstract

**OBJECTIVE:**

To confirm the diagram of the program’s impact pathways and *Estratégia Amamenta e Alimenta Brasil* (EAAB - Brazilian Breastfeeding and Complementary Feeding Strategy) core functions, and to determine indicators for evaluating EAAB.

**METHODS:**

This is a qualitative study within the field of implementation research. Data collection included two focus groups with EAAB implementers, document analysis, and literature review. The analysis included a review of the participants’ suggestions and two stages of reviewing the diagram and the names of the impact pathways and core functions. Questions for evaluating the EAAB were then constructed based on the confirmed diagram. The indicators of the RE-AIM framework (reach, effectiveness, adoption, implementation, maintenance) were adapted to the EAAB context. The evaluation questions were revised according to the RE-AIM domains and, finally, indicators were determined for each evaluation question.

**RESULTS:**

This study advanced the analysis of EAAB implementation, defining 22 indicators for its monitoring and evaluation. Most of the indicators are already used in implementation, however, the analysis with the RE-AIM framework allowed the indicators to be updated to be more specific, measurable, and relevant to the desired objectives.

**CONCLUSION:**

The results of the study support the pragmatic application of RE-AIM evaluation in health programs and encourage the planning of evaluation indicators for other child health and development programs in primary care.

## INTRODUCTION

In Brazil, primary care is the population’s gateway to the Unified Health System (SUS), which offers universal access to a network of care to protect, promote, and support health^1^. A large number of families are served in primary care: in 2020, 54,105 health teams worked in more than 44,000 basic health units (UBS) and served 76.1% of Brazil’s population, equivalent to more than 159.9 million people^2,3^. Adequate and healthy infant feeding is one of the health guidelines promoted in primary care. Data from the *Estudo Nacional de Alimentação e Nutrição Infantil* (Enani-2019 - Brazilian National Survey on Child Nutrition) showed that 45.8% of Brazilian children under six months were exclusively breastfed and 86.3% between six and eight months were being introduced to food^4,5^. The prevalence of overweight in children under five was 7.0%, while 18.3% of children were at risk of overweight^6^. Raising breastfeeding rates and improving the quality of food introduction in the first years of life is key to combating overweight and promoting children’s development to its full potential. To achieve these outcomes, the Brazilian Ministry of Health has been implementing the *Estratégia Amamenta e Alimenta Brasil* (EAAB - Brazilian Breastfeeding and Complementary Feeding Strategy) in primary care since 2013^7^.

The EAAB aims to qualify actions to promote and support breastfeeding and complementary feeding practices in the UBS. EAAB is implemented with multi-level governance by managers in the Ministry of Health and coordinators in all states and municipalities in Brazil^7,8^. Based on the cascade of continuing education for primary care professionals, the EAAB trains tutors to lead workshops with health teams in the UBS. The teams develop an action plan to help families with children under two. As a result, EAAB supports the training of professionals in the UBS and promotes better monitoring of food consumption markers for children under two^9,10^.

By 2018, more than 5,500 tutors had been trained, and more than 3,100 workshops had been held at the UBS^11^. Considering that there were more than 50,000 health teams in the country^3^, these results showed that the expansion of the EAAB was less than expected. The Ministry of Health therefore began a project to strengthen implementation with strategies on a national scale in four areas: support for coordinators; expansion of tutor training; monitoring of implementation in the municipalities; and research and evaluation^12^. As part of the research and evaluation axis, an analysis of the pathways to achieve the impact of the EAAB was conducted. Researchers interactively developed a diagram of the program’s impact pathway theory and analyzed barriers and facilitators to implementation. The results documented six critical control points of EAAB implementation, assumptions, facilitators, and contextual factors, described in another publication^8^. The analysis of these elements pointed to seven implementation core functions, i.e. planned activities that need to be followed to ensure successful implementation on a large scale^8^.

Experts recommend that after conducting the analysis of the program’s impact pathways diagram, it is important to consider implementing a robust impact evaluation system that allows for an objective understanding of whether the program is achieving its stated goals and whether it is following the pathways described in the diagram^13^. The choice of indicators should be informed by the analysis of the program’s impact pathways and can use conceptual frameworks that support the operationalization of the indicators^13^. The RE-AIM evaluation framework (reach, effectiveness, adoption, implementation, maintenance), for example, has a long history of use for planning and evaluating health programs^14^. In summary, the “reach” domain assesses how far the program reaches the beneficiary population; the “effectiveness” domain analyzes the benefits produced for the beneficiary population; the “adoption” domain verifies where and who adopts the program, with a greater focus on implementers, communities, organizations, and systems; the “implementation” domain assesses the consistency of implementation, costs, and adaptations in the delivery of actions; finally, the “maintenance” domain analyzes the sustainability of results over time, both for the beneficiary population and at the organizational level^14,15^. In this type of analysis, it is important to look at the indicators already in use by the programs, which can be consulted by reviewing the literature and discussing them with program workers and decision-makers. It is also useful to suggest new indicators that are needed, using evaluation models and frameworks to facilitate the systematized identification of indicators of implementation, effectiveness, and sustainability^16^.

Investment in EAAB implementation research in recent years has helped to clarify the pathways and activities central to its successful implementation. In order to advance the analysis of EAAB implementation, this study aimed to 1) confirm the diagram of program impact pathways and EAAB core functions, and 2) define monitoring and evaluation indicators to compose a robust EAAB evaluation scheme based on the RE-AIM framework.

## METHODS

The description of the methodological steps was guided by the Standards for Reporting Implementation Studies (StaRI)^17^ and consolidated criteria for reporting qualitative research (COREQ)^18^.

### Study Design

**Figure 1:**
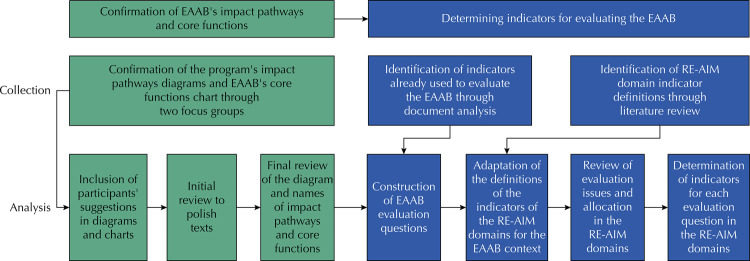
Methodological stages of data collection and analysis.

EAAB: Estratégia Amamenta e Alimenta Brasil (EAAB - Brazilian Breastfeeding and Complementary Feeding Strategy); RE-AIM: Reach, Effectiveness, Adoption, Implementation, Maintenance.

This is a qualitative observational study within the field of implementation research. Data collection included two focus groups with EAAB implementers, document analysis, and literature review. Figure 1 describes the stages of data collection and analysis in the two phases of the study: 1) confirming the impact pathways and core functions of the EAAB, and 2) determining indicators for evaluating the EAAB. This study was approved by the Research Ethics Committee of the School of Public Health at the University of São Paulo (USP), under protocol number 15184019.2.0000.5421. All participants signed an Informed Consent Form to register their intention to participate.

### Confirmation of EAAB Impact Paths and Core Functions

#### Selection of Participants

Key informants implementing the EAAB in the Ministry of Health, states, and municipalities in the country’s five macro-regions (North, Northeast, Midwest, Southeast, and South) were selected. A non-probabilistic purposive sample was selected. In order to capture the perspectives of different implementers, key informants who had already taken part in the study analyzing the program’s impact pathways were excluded^8^. The inclusion criteria and selected participants are described in Chart 1. States and municipalities with advanced stages of implementation were selected because they had more experience in implementing workshops and certifying UBS. The people selected were invited by e-mail to take part in the study. Only one municipality in the Northeast region was unavailable at the time, so key informants from the second municipality with the most advanced stage of EAAB in that region were invited.

**Chart 1:** Inclusion criteria and focus group participants.

Place of work	Inclusion criteria	Participants
Federal	Up to two professionals responsible for coordinating the EAAB in the Ministry of Health, who have been working on the EAAB for at least six months.	Total federal coordinators (n = 2) General Coordination of Food and Nutrition (n = 1) General Coordination of Perinatal Health and Breastfeeding (n = 1)
States	One state per macro-region with the highest proportion of municipalities that held EAAB workshops between 2013 and 2022. Up to two professionals per state, who have worked for at least six months as EAAB coordinators.	Total state coordinators (n = 7) North, Roraima (n = 1) Northeast, Ceará (n = 1) Midwest, Mato Grosso do Sul (n = 2) Southeast, Espírito Santo (n = 1) Sul, Rio Grande do Sul (n = 2)
Municipalities	One municipality per macro-region, with the highest number of certified basic health units. A professional who has worked for at least six months as the EAAB coordinator in the municipality.	Total municipal coordinators (n = 4) North, Manaus (n = 1) Midwest, Brasília (n = 1) Southeast, Ribeirão Preto (n = 1) South, Gravataí (n = 1)
	A professional EAAB tutor appointed by the municipal coordinator, with at least six months’ experience and who has held a workshop in a basic health unit.	Total of tutors (n = 2) Midwest, Brasília (n = 1) Southeast, Ribeirão Preto (n = 1)

EAAB: *Estratégia Amamenta e Alimenta Brasil* (EAAB - Brazilian Breastfeeding and Complementary Feeding Strategy); UBS: basic health unit.

#### Relations with Participants

Two authors, D.S.M. and S.I.V., were part of the national project to strengthen the EAAB and participated in remote meetings with EAAB implementers in states and municipalities across the country between 2020 and 2022^12^.

#### Data Collection

Two online focus groups were held in November 2022, one group with coordinators from the Ministry of Health and states and another group with municipal coordinators and tutors. A total of nine people took part in the first group and six people in the second, as well as a moderator (D.S.M.), an observer (S.I.V.), and a technical supporter (C.B.S.) experienced in qualitative methodology. A discussion guide was used, drawn up by a co-author (D.S.M) and revised by three co-authors (D.S.M., S.I.V., and G.B.). The groups were conducted in two stages: 1) to confirm the pathways diagram, and 2) to confirm the core functions. To make it easier to confirm the activities described in the EAAB program impact pathway diagram identified in a previous study^9^, the decision was made to adapt the information into six diagrams: 1) pathways for EAAB coordination and funding, 2) pathways for tutor training, 3) pathways for tutor support for UBS teams, 4) pathways for EAAB monitoring, 5) pathways for certification criteria, and 6) pathways for the UBS certification process. The first four diagrams of the EAAB program’s impact pathways were presented, and diagrams 5 and 6 were not presented because they were activities that were temporarily suspended in the implementation of EAAB at the time of the research. Secondly, a chart of the core functions of the EAAB was presented: 1) the presence of coordinators at the three levels of government and interfederative and intersectoral coordination; 2) the allocation of resources for the implementation of the EAAB; 3) the planning of implementation at the three levels of government; 4) the establishment of a group of national facilitators, the training of tutors and of UBS professionals; 5) the development of activities to obtain certification; 6) the monitoring and evaluation of implementation; 7) the dissemination of the EAAB. After the presentation of each diagram and the chart of core functions, the participants confirmed whether the descriptions were adequate or needed to be modified.

## Data Analysis

The notes and audio and video recordings of the two focus groups were revised by the first author to highlight the suggestions made by the key informants in the diagrams of the program’s impact pathways and EAAB’s core functions. Two co-authors (D.S.M. and S.I.V.) once again reviewed the materials to polish the texts. Then, three co-authors (D.S.M., S.I.V., and G.B.) organized the formatting of the final diagram and the names of the EAAB implementation pathways and core functions.

## Determining Indicators for Monitoring and Evaluating the EAAB

### Documentary Analysis

A document analysis was carried out to identify the indicators already used to monitor and evaluate the EAAB. We consulted the EAAB Implementation Manual^7^, the monitoring reports for the project to strengthen the EAAB in 2021 and 2022^12^, Ordinance No. 1.124 of May 19, 2022 (which established new indicators for monitoring the EAAB)^19^, and the primary care monitoring forms for recording food consumption and nutritional status markers and individual and collective activities in the UBS^20^.

### Literature Review

The RE-AIM evaluation framework was chosen to support the determination of EAAB evaluation indicators because it has a long history of use for planning and evaluating health programs^14^. A literature review was conducted to identify definitions of the RE-AIM domains with the aim of adapting them to evaluate EAAB. Peer-reviewed articles, supplementary texts, and official checklists available on the “https://re-aim.org/assessments/” website^21^ were included.

## Analysis

**Figure 2:**
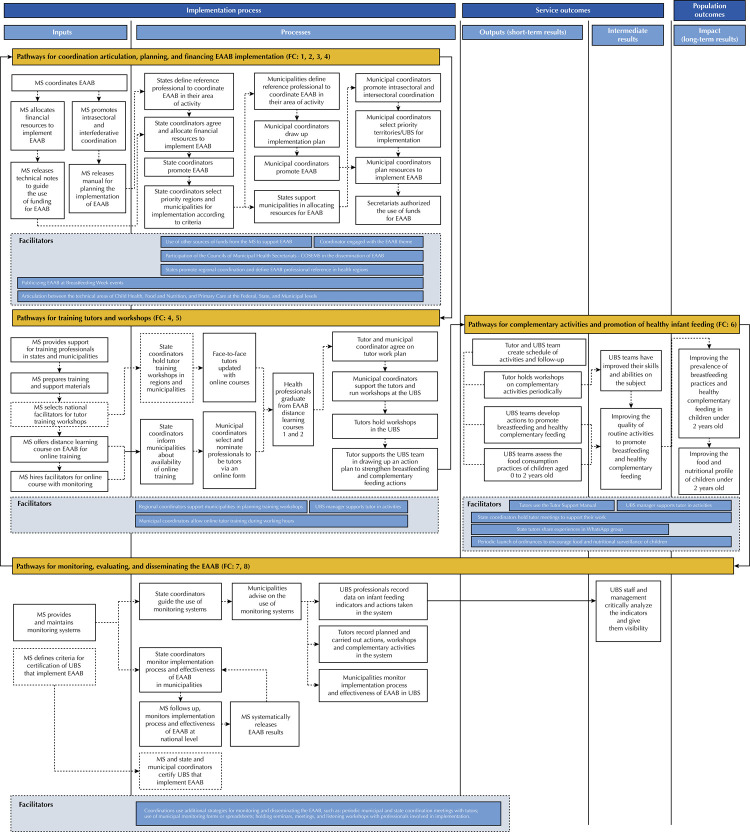
Diagram of EAAB program impact pathways confirmed and revised.

EAAB: *Estratégia Amamenta e Alimenta Brasil* (EABB - Brazilian Breastfeeding and Complementary Feeding Strategy); MS: Ministry of Health; UBS: basic health unit; FC: core function. FC: 1) Coordination, 2) Political and legislative support, 3) Financing and resources, 4) Planning and goals, 5) Training and workshops, 6) Delivery and promotion, 7) Research, monitoring, and evaluation, 8) Social communication.

The confirmed EAAB impact pathways diagram (Figure 2) and the indicators used to evaluate EAAB were consulted to guide the construction of EAAB evaluation questions. The evaluation questions were drafted in an interactive discussion process considering three main structures of the diagram: the implementation process, the outcomes in the service, and the outcomes in the population. Then, in an interactive discussion process, the authors adapted the definitions of the RE-AIM implementation outcome indicators to the EAAB context. The evaluation questions were then revised and allocated to the RE-AIM domains in the following order: adoption, implementation, reach, effectiveness, and maintenance. Finally, the indicators already used in the EAAB were reorganized, some grouped, others excluded or adapted, and so indicators were defined for each evaluation question and organized into levels: organizational (states, municipalities, and UBS), professional (tutors and health teams), and individual (beneficiary target population)^22^.

Dashed boxes: activities in the process of being revised for implementation in 2023.

## RESULTS

### Confirmation of EAAB Impact Pathways and Core Functions

The diagram of the EAAB program’s impact pathways after confirmation and review (Figure 2) is organized into three structures: “implementation process”, “service outcomes”, and “population outcomes”. Within these structures, the activities of “inputs”, “processes”, “outputs” (which include short-term and intermediate results), and “impacts”, which are the expected long-term results, are highlighted. The diagram then details four impact pathways: 1) pathways for coordination articulation, planning, and financing EAAB implementation; 2) pathways for training tutors and workshops; 3) pathways for complementary activities and promotion of healthy infant feeding; and 4) pathways for monitoring, evaluating, and disseminating the EAAB. Finally, the facilitators at the bottom of each pathway diagram are presented, which are activities or contexts that facilitate successful implementation.

Participants agreed on the definitions of the seven EAAB core functions. In order to make the names of the core functions more objective without compromising their content, the core functions were reorganized into eight items: 1) coordination, 2) political and legislative support, 3) financing and resources, 4) planning and goals, 5) training and workshops, 6) delivery and promotion, 7) research, monitoring and evaluation, and 8) social communication. This reorganization was based on the Breastfeeding Gear Model for programs in low- and middle-income countries^25^. The EAAB’s core functions are structural objectives and important activities for successful implementation, so they have been incorporated into the diagrams of the four impact pathways, as shown in Figure 2.

### Determining Indicators for Monitoring and Evaluating the EAAB

Eleven indicators were identified in the EAAB Implementation Manual, eleven in the EAAB strengthening project reports, and three in Ordinance No. 1.124/2022. Most of the indicators have been retained with adaptations to their description to make them more specific and measurable. Only three of them were excluded because they were specific activities in the certification criteria for actions in the UBS. A new indicator was added, which deals with assessing the stages of implementation of the EAAB. As a result, 22 indicators were determined to assess the activities that are in place in the implementation of the EAAB. Chart 2 describes: the definition of the RE-AIM indicators adapted for evaluating the EAAB, the evaluation questions, the evaluation indicators, and the data sources. In the “adoption” domain, four indicators were determined relating to the training of tutors and the presence of coordinators in the states and municipalities. In the “implementation” domain, seven indicators were determined relating to financial resources, the stage of implementation, and the consistency of implementation for the protection, promotion, and support of healthy infant feeding in the UBS. In the “reach” domain, three indicators were determined, focusing on the coverage of the target population of children under two years of age with EAAB actions. To assess the “effectiveness” domain, two indicators were determined: the percentage of records and the annual prevalence of infant feeding markers. Finally, in the “maintenance” domain, six indicators were determined to assess the institutionalization of the implementation of the EAAB in the municipalities, the continuity of the tutors’ work, and the continuous improvement of the effectiveness indicators.

**Chart 2:** Definitions of the RE-AIM domains for evaluating the *Estratégia Amamenta e Alimenta Brasil* (EAAB - Brazilian Breastfeeding and Complementary Feeding Strategy) and monitoring and evaluation indicators.

RE-AIM domains	Evaluation level	Definition for EAAB evaluation	EAAB assessment questions	Evaluation Indicators	Data source
Adoption (Where and who adopts)	Organizational (states and municipalities)	This refers to the percentage of states and municipalities with coordinators responsible for implementing and maintaining the EAAB.	What is the proportion of states and municipalities in the country with a professional acting as EAAB implementation coordinator?	Percentage of states with professionals acting as EAAB implementation coordinators in relation to the total number of states	Online form
				Percentage of municipalities with professionals working as EAAB implementation coordinators in relation to the total number of municipalities	
		This refers to the percentage of municipalities that have trained tutors.	What is the proportion of municipalities in the country with trained tutors?	Percentage of municipalities with trained tutors in relation to the total number of municipalities	EAAB management system
	Professional tutors	Refers to the number of tutors responsible for implementing and maintaining the EAAB.	Does the number of EAAB tutors in the municipality meet planning expectations?	Number of tutors in the municipality in relation to the number of planned tutors	EAAB management system
Implementation (How, and how consistent is the implementation)	Organizational (municipalities and UBS)	It refers to the financial resources for implementation and the stage of implementation of the EAAB in the country.	What is the proportion of municipalities in the country that are using financial resources (public resources to fund food and nutrition actions) to implement EAAB in UBS?	Percentage of municipalities that have allocated financial resources for the implementation of EAAB actions in relation to the total number of municipalities	Online form
			What proportion of UBS in the country are at an intermediate or advanced stage of EAAB implementation?	Percentage of UBS in the country at an intermediate stage of implementation (with a workshop)	EAAB management system
				Percentage of UBS in the country at an advanced stage of implementation (with certification)	
	Health team professionals	It refers to the consistency of the implementation of the EAAB for the protection, promotion, and support of healthy infant feeding in the UBS.	What proportion of health teams have an EAAB action plan?	Percentage of health teams with an EAAB action plan in relation to the total number of health teams per municipality	EAAB management system
			What proportion of health teams are developing systematic collective actions to promote breastfeeding and healthy complementary feeding?	Percentage of health teams with records of collective actions for health education with the beneficiary population of children aged 0 to 3 in relation to the total number of health teams per municipality	EAAB management system
					Primary Care Information System in force
			What proportion of health teams are monitoring the food consumption and nutritional status markers of children under two?	Number of health teams with records of food consumption markers for children aged 0-23 months in relation to the total number of health teams per municipality	Primary Care Information System in force
				Number of health teams recording the nutritional status of children aged 0-23 months in relation to the total number of health teams per municipality	
Reach (Who and how much is reached)	Individual (beneficiary population)	This refers to the percentage of the beneficiary population that took part in individual or collective actions to promote breastfeeding and complementary feeding and the coverage of feeding markers for children under two.	What is the coverage of children under the age of two who are being followed up with individual and collective actions at the UBS?	Percentage of children under the age of two monitored at the UBS in individual care in the municipality in relation to the total population of children under the age of two in the municipality	Primary Care Information System in force
				Percentage of children under the age of two monitored at the UBS in collective care in the municipality in relation to the total population of children under the age of two in the municipality	
			What is the coverage of children under the age of two who are having food consumption markers recorded at the UBS?	Percentage of children under two with food consumption markers recorded in relation to the total population of children under two in the municipality	
Effectiveness (what are the benefits)	Individual (beneficiary population)	This refers to the long-term outcome (impact), the extent to which the implementation of the EAAB affects the improvement in the recording, and prevalence of breastfeeding and complementary feeding indicators for children under two years of age.	Has there been an improvement in the recording and prevalence of feeding markers for children under two in the municipalities that have implemented the EAAB?	Percentage of food consumption markers recorded for children aged 0 to 23 months in relation to the previous year by health team	Primary Care Information System in force
				Prevalence of food consumption markers in children aged 0 to 23 months in relation to the previous year by health team	
Maintenance (When and for how long there are results)	Organizational (municipalities)	This refers to the institutionalization of EAAB implementation in the municipalities.	What proportion of municipalities in the country have an EAAB implementation plan?	Percentage of municipalities with an EAAB implementation plan for the current year in relation to the total number of municipalities	Online form
			What proportion of municipalities in the country have included EAAB in their municipal health plan?	Percentage of municipalities that have included EAAB in the municipal health plan in relation to the total number of municipalities	
		This refers to the continuity of implementation through the tutors’ work in the long term.	What is the proportion of municipalities with active tutors in the last year?	Percentage of municipalities with tutors who registered complementary activities at the UBS in the last six months in relation to the total number of municipalities in the country	
	Professional tutors		What is the proportion of active tutors per municipality?	Percentage of tutors who registered a complementary activity at the UBS in the last six months in relation to the total number of tutors trained in the municipality	
	Individual (beneficiary population)	It refers to the continuous improvement of the recording and prevalence of infant feeding markers in the long term.	Have the municipalities that have implemented the EAAB shown positive annual results in the recording and prevalence of infant feeding markers?	Percentage of municipalities that showed an improvement in the percentage of food consumption markers recorded for children aged 0 to 23 months in the last year in relation to the total number of municipalities	Primary Care Information Systems
				Percentage of municipalities that showed an improvement in the prevalence of food consumption markers for children aged 0 to 23 months in the last year in relation to the total number of municipalities	

Source: adapted from Melo et al.^9^, Glasgow et al.^18,19^; Holtrop et al.^20^

RE-AIM: reach, effectiveness, adoption, implementation, maintenance; UBS: basic health unit.

## DISCUSSION

This study advanced the analysis of the EAAB program impact pathways, interactively confirming the the activities and core functions of implementation with implementers from different levels and locations. The results of the initial analysis and the RE-AIM evaluation framework guided the unprecedented determination of indicators of EAAB adoption, implementation, reach, effectiveness, and maintenance.

Interactive confirmation of the program’s impact pathway diagram is described in the literature as an important strategy for health program coordinators to have a common understanding of program activities from the macro to the local level^13,26,27^. In line with the results described in other studies^26,27^, in this study the process of interaction between researchers and implementers helped to confirm the core activities of the program and gain an understanding of the indicators for measuring the objectives.

This study contributed with adjustments to the definitions of impact pathways, core functions, and indicators, aligning them with the taxonomy recommended in implementation science^28^. Thus, the 22 indicators proposed make it possible to monitor and evaluate implementation processes (existence of coordinators and training of tutors), outcomes in the service (operationalization of workshops and actions in the UBS), and outcomes in the population (registration and improvement of breastfeeding and complementary feeding markers). Conceptualizing and measuring implementation results, as presented in our research, is a critical step for understanding implementation processes, for increasing effectiveness in implementation research, and for opening avenues for comparative studies on implementation strategies^29^.

This study presented a comprehensive description of the methodological step-by-step process for determining indicators for the EAAB. This is an important contribution to the literature, as there is a lack of publications detailing the methods used to determine evaluation indicators based on the analysis of the program’s impact pathways^26,27^. In our study, the use of the RE-AIM evaluation framework to guide the definition of indicators ensured the robustness of the methods and results and confirmed the usefulness and applicability of this framework to guide the evaluation of large-scale programs such as the EAAB^30^.

The RE-AIM framework is a useful tool for determining adjustments and setting goals during the process of implementing health service improvement projects^31^. However, the studies are predominantly from high-income countries and the application of the framework has been more pragmatic, i.e. not all RE-AIM domains are usually assessed^32^. Studies have shown that, during program planning, most implementers focus more on the “reach” and “implementation” domains and less on the “effectiveness” and “maintenance” domains^31,33^, the latter being considered by members as the least important for proposing adjustments during project implementation. The present study fills these gaps by determining indicators for the five RE-AIM domains to plan the evaluation of a large-scale child nutrition promotion program in a middle-income country.

Indicators were defined to monitor and evaluate the EAAB, most of which were already being used. The analysis made it possible to update the indicators to be more specific, i.e. clearer to capture the desired changes in key outcomes, as recommended in the literature^13^. Experts recommend that the indicators informed by the analysis of the program’s impact pathways should be specific, measurable, achievable, relevant, and time-bound, noting whether they are already in use by the program or whether they are planned^13^. One example was updating the indicator “number of tutors trained” to “number of tutors in the municipality in relation to the number of tutors planned in the implementation plan”, so the indicator is more specific to adoption at the municipal level and measures the existence of a tutor training plan.

Some EAAB indicators were specific to certification criteria that were under review by the Ministry of Health. A previous study showed that UBS teams found it difficult to organize and send supporting documents for certain activities for certification, and the Ministry of Health had an overload of tasks which delayed analysis of the documents sent^8^. Therefore, it was justified to keep indicators of the certification criteria in the “implementation” domain that were measurable (quantitative and with tracking of progress over time) and achievable (realistically measurable with the data and resources available)^34^.

Aoki et al.^35^ used 14 indicators based on the RE-AIM framework to assess the stage of implementation of a maternal and child health promotion program in health institutions in Angola. The institutions were classified into optimal or suboptimal implementation groups, while interviews made it possible to complement the assessment and identify barriers and facilitators to implementation. In contrast, EAAB first underwent an analysis of barriers and facilitators to implementation and had its implementation strategies documented. This research generated positive results, especially in multilevel governance and tutor training^8,12^. It is understood that the indicators determined in this study could be used in the future to assess stages of implementation and identify priority municipalities for monitoring both the quality and effectiveness of EAAB implementation.

A strength of the indicator framework presented in this study is that it favors the application of the RE-AIM framework in the real world, agreeing with experts in the field who encourage the expansion of the use of RE-AIM in community-based programs^31^. In Brazil, few studies have used the RE-AIM framework to evaluate large-scale health programs and policies^36,37^. It is known that funding for the application of RE-AIM is often dependent on external program resources, such as national agencies, cooperation between academics, and health services and foundations^33^. This study was conducted as part of doctoral research funded by a national research agency. It was therefore possible to devote time to an in-depth analysis of the program’s impact pathways and RE-AIM domains and to propose a systematic EAAB evaluation framework. This highlights the importance of cooperation between academia and decision-makers in public policy and complex health programs, in order to make the pragmatic application of RE-AIM feasible.

This study has limitations and strengths. The data collected in the focus groups is limited to the perceptions of the participants who attended the meetings, and who were only from municipalities with an advanced implementation context. The second focus group saw the absence of the municipal coordinator and tutor from the North-East region and the absence of tutors from the North and South regions. On the other hand, in the interactive process of confirming the impact pathways diagram, it was observed that most of the suggestions from the second group were similar to those from the first group, demonstrating the saturation and robustness of the results presented. Some of the indicators determined for the EAAB require the Ministry of Health to periodically send online forms to municipalities, as they are not available in official primary health care information systems. We understand that future adaptations to the data sources for these indicators may be necessary. Despite this limitation, we believe that these indicators should be maintained as part of the EAAB evaluation system, as they refer to the existence of coordinators, funding, and the implementation plan, which are critical points for control, identified in a previous study analyzing the implementation of the EAAB^8^.

## CONCLUSIONS

This study advanced the analysis of EAAB implementation by determining indicators for evaluation with reference to implementation research theories and frameworks. The theory of impact pathways and the confirmed core functions act as gears to guide the implementation of EAAB on a large scale in a consistent manner and achieve successful implementation. The knowledge gained through interaction with EAAB coordinators from the country’s five macro-regions supports the generalization of the results. Indicators were defined to monitor and evaluate the EAAB which, for the most part, were already being used in the implementation. However, the analysis with the RE-AIM framework allowed the indicators to be updated to be more specific, measurable and relevant to the desired objectives. Overall, the results of the study support the pragmatic application of RE-AIM evaluation in health programs and encourage the planning of evaluation indicators for other child health and development programs in primary care.
