# Supplementary material for: Determining RE-AIM indicators for evaluating the Estratégia Amamenta e Alimenta Brasil (EAAB – Brazilian Breastfeeding and Complementary Feeding Strategy)
Source: Rev Saude Publica. 2024 Sep 16;58:43. doi: 10.11606/s1518-8787.2024058005875 (PMC11548908; doi:10.11606/s1518-8787.2024058005875)
Supplement: Translation

# Translation: Determinação de indicadores RE-AIM para avaliação da Estratégia Amamenta e Alimenta Brasil

**Authors:** Daiane Sousa Melo, Sonia Isoyama Venancio, Gabriela Buccini

**Affiliations:** I Brasil Universidade de São Paulo. Faculdade de Saúde Pública. Departamento de Nutrição. São Paulo, SP, Brasil; II Brasil Ministério da Saúde. Coordenação de Atenção à Saúde da Criança e do Adolescente. Brasília, DF, Brasil; III USA University of Nevada Las Vegas. School of Public Health. Department of Social and Behavioral Health. Las Vegas, USA

**Keywords:** Aleitamento Materno, Fenômenos Fisiológicos da Nutrição do Lactente, Atenção Primária à Saúde, Ciência da Implementação

## Abstract

**OBJETIVO:**

Confirmar o diagrama dos caminhos de impacto do programa e funções centrais da Estratégia Amamenta e Alimenta Brasil (EAAB) e determinar indicadores para avaliação da EAAB.

**MÉTODOS:**

Trata-se de um estudo qualitativo, inserido no campo da pesquisa de implementação. A coleta de dados incluiu dois grupos focais com implementadores da EAAB, análise documental e revisão da literatura. A análise incluiu revisão das sugestões dos participantes e duas etapas de revisão do diagrama e dos nomes dos caminhos de impacto e funções centrais. Em seguida, foram construídas questões para a avaliação da EAAB com base no diagrama confirmado. Os indicadores do *framework* RE-AIM (*reach, effectiveness, adoption, implementation, maintenance*) foram adaptados para o contexto da EAAB. As questões de avaliação foram revisadas segundo domínios RE-AIM e, por fim, foram determinados indicadores para cada questão de avaliação.

**RESULTADOS:**

Este estudo avançou na análise de implementação da EAAB, definindo 22 indicadores para seu monitoramento e avaliação. A maioria dos indicadores já são utilizados na implementação, entretanto, a análise com o *framework* RE-AIM permitiu atualizar os indicadores para serem mais específicos, mensuráveis e relevantes para os objetivos desejados.

**CONCLUSÃO:**

Os resultados do estudo apoiam a aplicação pragmática da avaliação RE-AIM em programas de saúde e incentivam o planejamento de indicadores de avaliação de outros programas de saúde e desenvolvimento infantil na atenção primária.

## INTRODUÇÃO

No Brasil, a atenção primária é a porta de entrada da população ao Sistema Único de Saúde (SUS), o qual oferece acesso universal a uma rede de cuidados para proteção, promoção e apoio à saúde^1^. Grande número de famílias são atendidas na atenção primária: em 2020, 54.105 equipes de saúde atuavam em mais de 44 mil unidades básicas de saúde (UBS) e atendiam 76,1% da população do Brasil, equivalente a mais de 159,9 milhões de pessoas^2,3^. A alimentação infantil adequada e saudável é uma das diretrizes de saúde promovidas na atenção primária. Dados do Estudo Nacional de Alimentação e Nutrição Infantil (Enani-2019) mostraram 45,8% das crianças brasileiras menores de seis meses em aleitamento materno exclusivo e 86,3% entre seis a oito meses em introdução alimentar^4,5^. A prevalência de sobrepeso em crianças menores de cinco anos era de 7,0%, enquanto 18,3% das crianças estavam em risco de sobrepeso^6^. Elevar os índices de aleitamento materno e melhorar a qualidade da introdução de alimentos nos primeiros anos de vida é fundamental para combater o excesso de peso e promover o desenvolvimento das crianças em seu potencial máximo. Para obter esses desfechos, o Ministério da Saúde do Brasil implementa, desde 2013, a Estratégia Amamenta e Alimenta Brasil (EAAB) na atenção primária^7^.

A EAAB visa qualificar ações de promoção e apoio ao aleitamento materno e práticas de alimentação complementar nas UBS. A EAAB é implementada com uma governança multinível por gestores no Ministério da Saúde e coordenadores em todos os estados e municípios do Brasil^7,8^. A partir da educação permanente em cascata de profissionais da atenção primária, a EAAB forma tutores para liderarem oficinas de trabalho com as equipes de saúde nas UBS. As equipes desenvolvem um plano de ação para atender famílias de crianças menores de dois anos. Como resultado, a EAAB apoia a capacitação dos profissionais nas UBS e promove melhor acompanhamento dos marcadores do consumo alimentar das crianças menores de dois anos^9,10^.

Em 2018, estavam formados mais de 5,5 mil tutores, que realizaram mais de 3,1 mil oficinas de trabalho nas UBS^11^. Considerando que havia mais de 50 mil equipes de saúde no país^3^, esses resultados demonstraram que expansão da EAAB estava aquém do esperado. Assim, o Ministério da Saúde iniciou um projeto de fortalecimento da implementação com estratégias em escala nacional em quatro eixos: apoio aos coordenadores; expansão da formação de tutores; monitoramento da implementação nos municípios; e pesquisa e avaliação^12^. Como parte do eixo pesquisa e avaliação, foi conduzida uma análise dos caminhos para alcançar o impacto da EAAB. Pesquisadores desenvolveram interativamente um diagrama da teoria dos caminhos de impacto do programa e analisaram barreiras e facilitadores para a implementação. Os resultados documentaram seis pontos críticos de controle da implementação da EAAB, premissas, facilitadores e fatores contextuais, descritos em outra publicação^8^. A análise desses elementos apontou sete funções centrais da implementação, ou seja, atividades planejadas que precisam ser seguidas para garantir a implementação bem-sucedida em larga escala^8^.

Especialistas recomendam que, após conduzir a análise do diagrama dos caminhos de impacto do programa, é importante considerar a implementação de um sistema sólido de avaliação de impacto que permita uma compreensão objetiva se o programa está atingindo as metas declaradas e se está seguindo os caminhos descritos no diagrama^13^. A escolha de indicadores deve ser informada pela análise dos caminhos de impacto do programa e pode utilizar estruturas/*frameworks* conceituais que apoiem a operacionalização dos indicadores^13^. O *framework* de avaliação RE-AIM (*reach, effectiveness, adoption, implementation, maintenance*), por exemplo, tem um longo histórico de uso para o planejamento e avaliação de programas de saúde^14^. Em síntese, o domínio “*reach*” avalia o quanto o programa alcança a população beneficiária; o domínio “*effectiveness*” analisa quais são os benefícios produzidos para a população beneficiária; o domínio “*adoption*” verifica onde e quem adota o programa, com maior foco nos implementadores, comunidades, organizações e sistemas; o domínio “*implementation*” afere a consistência da implementação, dos custos e das adaptações na entrega das ações; por fim, o domínio “*maintenance*” analisa a sustentabilidade dos resultados ao longo do tempo, tanto para a população beneficiária quanto no nível organizacional^14,15^. Nesse tipo de análise é importante observar os indicadores já em uso pelos programas, os quais podem ser consultados com revisão da literatura e discussão com trabalhadores e tomadores de decisão do programa. Também é útil sugerir novos indicadores necessários, utilizando modelos e estruturas de avaliação para facilitar a identificação sistematizada de indicadores de implementação, efetividade e sustentabilidade^16^.

O investimento em pesquisa de implementação da EAAB nos últimos anos ajudou a esclarecer os caminhos e as atividades centrais para o sucesso da sua implementação. Visando avançar na análise de implementação da EAAB, este estudo teve como objetivo: 1) confirmar o diagrama dos caminhos de impacto do programa e funções centrais da EAAB e 2) definir indicadores para monitoramento e avaliação para compor um esquema robusto de avaliação da EAAB com base no *framework* RE-AIM.

## MÉTODOS

A descrição das etapas metodológicas foi guiada pelo *Standards for Reporting Implementation Studies* (StaRI)^17^ e *Consolidated criteria for reporting qualitative research* (COREQ)^18^.

### Desenho do Estudo

Trata-se de um estudo observacional qualitativo, inserido no campo da pesquisa de implementação. A coleta de dados incluiu dois grupos focais com implementadores da EAAB, análise documental e revisão da literatura. A Figura 1 descreve as etapas da coleta e análise de dados nas duas fases do estudo: 1) confirmação dos caminhos de impacto e funções centrais da EAAB e 2) definição de indicadores para avaliação da EAAB. Esta pesquisa foi aprovada pelo Comitê de Ética em Pesquisa da Faculdade de Saúde Pública da Universidade de São Paulo (USP), sob protocolo de número 15184019.2.0000.5421. Todos os participantes assinaram um Termo de Consentimento Livre e Esclarecido para registrarem sua intenção de participação.

**Figura 1:** Etapas metodológicas de coleta e análise dos dados. EAAB: Estratégia Amamenta e Alimenta Brasil; RE-AIM: Reach, Effectiveness, Adoption, Implementation, Maintenance.

### Confirmação dos Caminhos de Impacto e Funções Centrais da EAAB

#### Seleção dos Participantes

Foram selecionados informantes-chave implementadores da EAAB no Ministério da Saúde, estados e municípios das cinco macrorregiões do país (Norte, Nordeste, Centro-Oeste, Sudeste e Sul). Foi selecionada uma amostra não probabilística intencional. Para captar as perspectivas de diferentes implementadores, foram excluídos informantes-chave que já haviam participado do estudo de análise dos caminhos de impacto do programa^8^. Os critérios de inclusão e participantes selecionados são descritos no Quadro 1. Foram selecionados estados e municípios com características de estágios avançados de implementação por terem mais experiência na implementação das oficinas de trabalho e certificação das UBS. As pessoas selecionadas foram convidadas por e-mail para participar da pesquisa. Apenas um município da região Nordeste não tinha disponibilidade na data, assim foram convidados informantes-chave do segundo município com estágio mais avançado da EAAB naquela região.

**Quadro 1:** Critérios de inclusão e participantes dos grupos focais.

Local de atuação	Critério de inclusão	Participantes
Federal	Até dois profissionais responsáveis pela coordenação da EAAB no Ministério da Saúde, atuando há pelo menos seis meses na EAAB.	Total de Coordenadores Federais (n=2) Coordenação-Geral de Alimentação e Nutrição (n = 1) Coordenação-Geral de Saúde Perinatal e de Aleitamento Materno (n = 1)
Estados	Um estado por macrorregião com maior proporção de municípios que realizaram oficinas de trabalho da EAAB entre os anos de 2013 e 2022. Até dois profissionais por estado, que trabalharam por pelo menos seis meses como coordenadores da EAAB.	Total de coordenadores estaduais (n = 7) Norte, Roraima (n = 1) Nordeste, Ceará (n = 1) Centro-Oeste, Mato Grosso do Sul (n = 2) Sudeste, Espírito Santo (n = 1) Sul, Rio Grande do Sul (n = 2)
Municípios	Um município por macrorregião, com maior número de unidades básicas de saúde certificadas. Um profissional que trabalhou por pelo menos seis meses como coordenador da EAAB no município.	Total de coordenadores municipais (n = 4) Norte, Manaus (n = 1) Centro-Oeste, Brasília (n = 1) Sudeste, Ribeirão Preto (n = 1) Sul, Gravataí (n = 1)
	Um profissional tutor da EAAB indicado pelo coordenador municipal, com pelo menos seis meses de atuação e que realizou oficina de trabalho em uma unidade básica de saúde.	Total de tutores (n = 2) Centro-Oeste, Brasília (n = 1) Sudeste, Ribeirão Preto (n = 1)

EAAB: Estratégia Amamenta e Alimenta Brasil; UBS: unidade básica de saúde.

#### Relações com os Participantes

Duas autoras, D.S.M. e S.I.V. fizeram parte do projeto nacional de fortalecimento da EAAB e participaram de reuniões remotas com implementadores da EAAB nos estados e municípios de todo o país, no período entre 2020 e 2022^12^.

#### Coleta dos Dados

Foram conduzidos dois grupos focais *online* em novembro de 2022, sendo um grupo com coordenadores do Ministério da Saúde e de estados, e outro grupo com coordenadores municipais e tutores. Ao total participaram do primeiro grupo nove pessoas e, do segundo grupo, seis pessoas, além de uma moderadora (D.S.M.), uma observadora (S.I.V) e uma apoiadora técnica (C.B.S.) experientes em metodologia qualitativa. Foi utilizado um guia de discussão, elaborado por uma coautora (D.S.M) e revisado por três coautoras (D.S.M., S.I.V. e G.B.). Os grupos foram conduzidos em dois momentos: 1) confirmar o diagrama dos caminhos e 2) confirmar as funções centrais. Para facilitar a confirmação das atividades descritas no diagrama dos caminhos de impacto do programa da EAAB identificado em estudo prévio^9^, optou-se por adaptar as informações em seis diagramas: 1) caminhos das articulações e financiamento da EAAB, 2) caminhos para a formação de tutores, 3) caminhos do apoio dos tutores às equipes das UBS, 4) caminhos para o monitoramento da EAAB, 5) caminhos dos critérios de certificação e 6) caminhos do processo de certificação das UBS. Foram apresentados os quatro primeiros diagramas dos caminhos de impacto do programa da EAAB, e os diagramas 5 e 6 não foram apresentados por se tratar de atividades temporariamente suspensas na implementação da EAAB no momento da pesquisa. Num segundo momento, foi apresentado um quadro das funções centrais da EAAB: 1) presença de coordenadores nos três níveis de governo e articulação interfederativa e intersetorial; 2) alocação de recursos para a implementação da EAAB; 3) planejamento da implementação nas três esferas de governo; 4) estabelecimento de um grupo de facilitadores nacionais, formação de tutores e dos profissionais das UBS; 5) desenvolvimento de atividades para obter a certificação; 6) monitoramento e avaliação da implementação; 7) disseminação da EAAB. Após a apresentação de cada diagrama e do quadro de funções centrais, os participantes confirmaram se as descrições estavam adequadas ou se necessitavam de modificações.

## Análise dos Dados

As anotações e os registros de áudio e vídeo dos dois grupos focais foram revisados pela primeira autora para destacar as sugestões apontadas pelos informantes-chave nos diagramas dos caminhos de impacto do programa e funções centrais da EAAB. Duas coautoras (D.S.M. e S.I.V.) revisaram mais uma vez os materiais para polir os textos. Em seguida, três coautoras (D.S.M., S.I.V. e G.B.) organizaram a formatação do diagrama final e os nomes dos caminhos da implementação da EAAB e das funções centrais.

## Determinação de Indicadores para Monitoramento e Avaliação da EAAB

### Análise Documental

Foi feita análise documental visando identificar os indicadores já utilizados para avaliar a EAAB. Foram consultados o Manual de Implementação da EAAB^7^, os relatórios de monitoramento do projeto de fortalecimento da EAAB em 2021 e 2022^12^, a Portaria n^o^ 1.124, de 19 de maio de 2022 (que estabeleceu novos indicadores para monitoramento da EAAB)^19^, e as fichas de monitoramento da atenção básica para registro de marcadores de consumo alimentar e estado nutricional e atividades individuais e coletivas nas UBS^20^.

### Revisão de Literatura

O *framework* de avaliação RE-AIM foi escolhido para apoiar a determinação dos indicadores de monitoramento e avaliação da EAAB por ter um longo histórico de uso para o planejamento e avaliação de programas de saúde^14^. Foi realizada revisão da literatura para identificar definições dos domínios RE-AIM com o objetivo de adaptá-las para avaliar a EAAB. Foram incluídos artigos revisados por pares, textos complementares e *checklists* oficiais disponíveis no website: https://re-aim.org/assessments/^21^.

## Análise

O diagrama confirmado dos caminhos de impacto da EAAB (Figura 2) e os indicadores utilizados para avaliar a EAAB foram consultados para guiar a construção de questões para avaliação da EAAB. As questões de avaliação foram rascunhadas em um processo interativo de discussões considerando três estruturas principais do diagrama: o processo de implementação, os desfechos no serviço e os desfechos na população. Em seguida, em um processo interativo de discussões, as autoras adaptaram as definições dos indicadores de desfechos de implementação RE-AIM para o contexto da EAAB. Então, as questões de avaliação foram revisadas e alocadas nos domínios do RE-AIM na ordem: adoção, implementação, alcance, efetividade e manutenção. Por fim, os indicadores já utilizados na EAAB foram reorganizados, alguns agrupados, outros excluídos ou adaptados, e assim foram definidos indicadores para cada questão de avaliação e organizados em níveis: organizacional (estados, municípios e UBS), dos profissionais (tutores e equipes de saúde), e individual (população alvo beneficiária)^22^.

**Figura 2:** Diagrama dos caminhos de impacto do programa da EAAB confirmado e revisado. EAAB: Estratégia Amamenta e Alimenta Brasil; MS: Ministério da Saúde; UBS: unidade básica de saúde; FC: função central. Nota: função central: 1) Coordenação, 2) Apoio político e legislativo, 3) Financiamento e recursos, 4) Planejamento e metas, 5) Treinamento e oficinas de trabalho, 6) Entrega e promoção, 7) Pesquisa, monitoramento e avaliação, 8) Comunicação social. Caixas tracejadas: atividades em processo de revisão na implementação no ano de 2023.

## RESULTADOS

### Confirmação dos Caminhos de Impacto e Funções Centrais da EAAB

O diagrama dos caminhos de impacto do programa da EAAB após confirmação e revisão (Figura 2) é organizado em três estruturas: “processo de implementação”, “desfechos no serviço” e “desfechos na população”. Dentro dessas estruturas são destacadas as atividades de “entradas”, “processos”, “saídas” (que incluem resultados de curto prazo e intermediários) e “impactos” os quais são os resultados esperados no longo prazo. Então são detalhados no diagrama quatro caminhos de impacto: 1) caminhos das articulações entre coordenadores, planejamento e financiamento da implementação EAAB; 2) caminhos do treinamento para formação de tutores e oficinas de trabalho; 3) caminhos das atividades complementares e promoção da alimentação infantil saudável; e 4) caminhos para o monitoramento, avaliação e disseminação da EAAB. Por fim, são apresentados os facilitadores na base do diagrama de cada caminho, que são atividades ou contextos que facilitam obter sucesso na implementação.

Os participantes concordaram com as definições das sete funções centrais da EAAB. Visando deixar os nomes das funções centrais mais objetivos, sem comprometer o seu conteúdo, as funções centrais foram reorganizadas em oito itens: 1) governança multinível, 2) apoio político e legislativo, 3) financiamento e recursos, 4) planejamento e metas, 5) treinamento e oficinas de trabalho, 6) entrega e promoção, 7) pesquisa, monitoramento e avaliação e 8) comunicação social. Essa reorganização teve como referência o modelo das engrenagens para o sucesso de programas de promoção da amamentação em países de baixa e média renda^25^. As funções centrais da EAAB são objetivos estruturais e atividades importantes para o sucesso da implementação, portanto, elas foram incorporadas aos diagramas dos quatro caminhos de impacto, como apresentado na Figura 2.

### Determinação de Indicadores para Monitoramento e Avaliação da EAAB

Foram identificados 11 indicadores no Manual de Implementação da EAAB, 11 nos relatórios do projeto de fortalecimento da EAAB e três na Portaria n^o^ 1.124/2022. A maioria dos indicadores foi mantida com adaptações em sua descrição para serem mais específicos e mensuráveis. Apenas três deles foram excluídos por se tratar de atividades específicas de critérios de certificação de ações nas UBS. Um novo indicador foi acrescentado, que se trata da avaliação dos estágios de implementação da EAAB. Como resultado, foram determinados 22 indicadores para avaliar as atividades que estão em vigor na implementação da EAAB. O Quadro 2 descreve: a definição dos indicadores RE-AIM adaptados para avaliação da EAAB, as questões para avaliação, os indicadores de avaliação e as fontes dos dados. No domínio “adoção” foram determinados quatro indicadores relacionados à formação de tutores e à presença de coordenadores nos estados e municípios. No domínio “implementação”, foram determinados sete indicadores referentes aos recursos financeiros, ao estágio de implementação e à consistência da implementação para a proteção, a promoção e o apoio da alimentação infantil saudável nas UBS. No domínio “alcance”, foram determinados três indicadores, focando na cobertura da população alvo de crianças menores de dois anos com as ações da EAAB. Para avaliar o domínio “efetividade” foram determinados dois indicadores do percentual de registro e da prevalência anual dos marcadores da alimentação infantil. Por fim, no domínio “manutenção” foram determinados seis indicadores que avaliam a institucionalização da implementação da EAAB nos municípios, a continuidade da atuação dos tutores e a contínua melhoria dos indicadores de efetividade.

**Quadro 2:** Definições dos domínios RE-AIM para avaliar a Estratégia Amamenta e Alimenta Brasil e indicadores de monitoramento e avaliação.

Domínios RE-AIM	Nível de avaliação	Definição para avaliação da EAAB	Questões para avaliação da EAAB	Indicadores de avaliação	Fonte de dados
Adoção (Onde e quem adota)	Organizacional (estados e municípios)	Refere-se ao percentual de estados e municípios com coordenadores responsáveis pela implementação e manutenção da EAAB.	Qual a proporção de estados e municípios no país com profissional atuando como coordenador da implementação da EAAB?	Percentual de estados com profissionais atuando como coordenador da implementação EAAB em relação ao total de estados	Formulário *online*
				Percentual de municípios com profissionais atuando como coordenador da implementação EAAB em relação ao total de municípios	
		Refere-se ao percentual dos municípios que fizeram formação de tutores.	Qual a proporção de municípios no país com tutores formados?	Percentual de municípios com tutores formados em relação ao total de municípios	Sistema de gerenciamento da EAAB
	Profissionais tutores	Refere-se ao número de tutores responsáveis pela implementação e manutenção da EAAB.	O número de tutores da EAAB no município atende as expectativas do planejamento?	Número de tutores no município em relação ao número de tutores planejados	Sistema de gerenciamento da EAAB
Implementação (Como, e quão consistente é a implementação)	Organizacional (municípios e UBS)	Refere-se aos recursos financeiros para a implementação e ao estágio de implementação da EAAB no país.	Qual a proporção de municípios no país que estão utilizando recursos financeiros (recursos públicos de custeio de ações de alimentação e nutrição) para implementar a EAAB nas UBS?	Percentual de municípios que alocaram recursos financeiros para a implementação de ações da EAAB em relação ao total de municípios	Formulário *online*
			Qual a proporção de UBS no país que estão em estágio de implementação intermediário ou avançado da implementação da EAAB?	Percentual de UBS no país com estágio de implementação intermediário (com oficina de trabalho)	Sistema de gerenciamento da EAAB
				Percentual de UBS no país com estágio de implementação avançado (com certificação)	
	Profissionais das equipes de saúde	Refere-se à consistência da implementação da EAAB para a proteção, a promoção e o apoio da alimentação infantil saudável nas UBS.	Qual a proporção de equipes de saúde com um plano de ação da EAAB?	Percentual de equipes de saúde com plano de ação da EAAB em relação ao total de equipes de saúde por município	Sistema de gerenciamento da EAAB
			Qual a proporção de equipes de saúde que estão desenvolvendo ações sistemáticas coletivas para promoção da amamentação e alimentação complementar saudável?	Percentual de equipes de saúde com registros das ações coletivas para educação em saúde com população beneficiária de crianças de 0 a 3 anos em relação ao total de equipes de saúde por município	Sistema de gerenciamento da EAAB
					Sistema de Informação da Atenção Básica vigente
			Qual a proporção de equipes de saúde que estão monitorando os marcadores de consumo alimentar e do estado nutricional das crianças menores de dois anos?	Número de equipes de saúde com registros dos marcadores de consumo alimentar de crianças de 0 a 23 meses em relação ao total de equipes de saúde por município	Sistema de Informação da Atenção Básica vigente
				Número de equipes de saúde com registro do estado nutricional de crianças de 0 a 23 meses em relação ao total de equipes de saúde por município	
Alcance (Quem e o quanto é alcançado)	Individual (população beneficiária)	Refere-se ao percentual da população beneficiária que participou das ações individuais ou coletivas para a promoção da amamentação e alimentação complementar e a cobertura dos marcadores de alimentação das crianças menores de dois anos.	Qual a cobertura de crianças menores de dois anos que estão sendo acompanhadas com ações individuais e coletivas nas UBS?	Percentual de crianças menores de dois anos acompanhadas nas UBS em atendimentos individuais no município em relação ao total da população de crianças menores de dois anos no município	Sistema de Informação da Atenção Básica vigente
				Percentual de crianças menores de dois anos acompanhadas nas UBS em atendimentos coletivos no município em relação ao total da população de crianças menores de dois anos no município	
			Qual a cobertura de crianças menores de dois anos que estão tendo os marcadores de consumo alimentar registrados nas UBS?	Percentual de crianças menores de dois anos com registro dos marcadores de consumo alimentar em relação ao total da população de crianças menores de dois anos no município	
Efetividade (Quais são os benefícios)	Individual (população beneficiária)	Refere-se ao desfecho de longo prazo (impacto), ao quanto a implementação da EAAB afeta a melhoria do registro e da prevalência de indicadores de amamentação e alimentação complementar das crianças menores de dois anos.	Houve melhora no registro e na prevalência dos marcadores de alimentação das crianças menores de dois anos nos municípios que implementaram a EAAB?	Percentual de registro dos marcadores de consumo alimentar de crianças de 0 a 23 meses em relação ao ano anterior por equipe de saúde	Sistema de Informação da Atenção Básica vigente
				Prevalência dos marcadores de consumo alimentar de crianças de 0 a 23 meses em relação ao ano anterior por equipe de saúde	
Manutenção (Quando e por quanto tempo há resultados)	Organizacional (municípios)	Refere-se à institucionalização da implementação da EAAB nos municípios.	Qual a proporção de municípios no país com um plano de implementação da EAAB?	Percentual de municípios com plano de implementação da EAAB para o ano vigente em relação ao total de municípios	Formulário *online*
			Qual a proporção de municípios no país que incluíram a EAAB no plano municipal de saúde?	Percentual de municípios que incluíram a EAAB no plano municipal de saúde em relação ao total de municípios	
		Refere-se à continuidade da implementação por meio da atuação dos tutores no longo prazo.	Qual a proporção de municípios com tutores ativos no último ano?	Percentual de municípios com tutores que registraram atividade complementar na UBS nos últimos seis meses em relação ao total de municípios no país	
	Profissionais tutores		Qual a proporção de tutores ativos por município?	Percentual de tutores que registraram atividade complementar na UBS nos últimos seis meses em relação ao total de tutores formados no município	
	Individual (população beneficiária)	Refere-se à contínua melhoria do registro e da prevalência dos marcadores de alimentação infantil no longo prazo.	Os municípios que implementaram a EAAB têm mostrado resultados anuais positivos no registro e na prevalência dos marcadores de alimentação infantil?	Percentual de municípios que apresentaram melhoria no percentual de registro dos marcadores de consumo alimentar de crianças de 0 a 23 meses no último ano em relação ao total de municípios	Sistemas de Informação da Atenção Primária
				Percentual de municípios que apresentaram melhoria na prevalência dos marcadores de consumo alimentar de crianças de 0 a 23 meses no último ano em relação ao total de municípios	

Fonte: adaptado de Melo et al.^9^, Glasgow et al.^18^^,^^19^; Holtrop et al.^20^

RE-AIM: reach, effectiveness, adoption, implementation, maintenance; EAAB: Estratégia Amamenta e Alimenta Brasil; UBS: unidade básica de saúde.

## DISCUSSÃO

Este estudo avançou na análise dos caminhos de impacto do programa da EAAB, confirmando interativamente as atividades e funções centrais da implementação com implementadores de diferentes níveis e locais de atuação. Os resultados da análise inicial e o *framework* de avaliação RE-AIM orientaram a determinação inédita de indicadores de adoção, implementação, alcance, efetividade e manutenção da EAAB.

A confirmação interativa do diagrama dos caminhos de impacto do programa é descrita na literatura como uma estratégia importante para que os coordenadores de programas de saúde tenham um conhecimento comum das atividades do programa do nível macro ao local^13,26,27^. Em consonância com resultados descritos em outros estudos^26,27^, na presente pesquisa o processo de interação entre pesquisadores e implementadores ajudou a confirmar as atividades centrais do programa e ter uma compreensão dos indicadores para mensurar os objetivos.

Este estudo contribuiu com ajustes nas definições dos caminhos de impacto, funções centrais e indicadores, alinhando a taxonomia recomendada na ciência da implementação^28^. Assim, os 22 indicadores propostos permitem monitorar e avaliar processos de implementação (existência de coordenadores e formação de tutores), desfechos no serviço (operacionalização das oficinas de trabalho e ações nas UBS) e desfechos na população (registro e melhoria dos marcadores da amamentação e alimentação complementar). Conceituar e medir os resultados da implementação, como apresentado em nossa pesquisa, é uma etapa crítica para a compreensão dos processos de implementação, para aumentar a efetividade na pesquisa sobre implementação, e para abrir caminhos para estudos comparativos sobre as estratégias de implementação^29^.

Este estudo apresentou uma descrição compreensiva do passo a passo metodológico para determinar indicadores para a EAAB. Essa é uma importante contribuição para a literatura, por ser observada uma escassez de publicações que demonstram o detalhamento dos métodos utilizados na determinação de indicadores de avaliação a partir da análise dos caminhos de impacto do programa^26,27^. Nesse sentido, em nosso estudo, a utilização do *framework* de avaliação RE-AIM para guiar a definição dos indicadores, garantiu robustez dos métodos e dos resultados e confirmou a utilidade e aplicabilidade desse *framework* para guiar a avaliação de programas em larga escala como a EAAB^30^.

O *framework* RE-AIM é uma ferramenta útil para determinar ajustes e estabelecer metas durante o processo de implementação de projetos de melhoria de serviços de saúde^31^. Entretanto, os estudos são predominantemente de países de alta renda e a aplicação do *framework* tem sido mais pragmática, ou seja, geralmente não são avaliados todos os domínios RE-AIM^32^. Estudos demonstraram que, durante o planejamento de programas, a maioria dos implementadores dão maior foco para os domínios “alcance” e “implementação” e menor atenção para os domínios “efetividade” e “manutenção”^31,33^, sendo esse último considerado pelos membros como o menos importante para propor ajustes durante a implementação dos projetos. O presente estudo preenche essas lacunas ao determinar indicadores dos cinco domínios RE-AIM para planejar a avaliação de um programa de promoção da alimentação infantil em larga escala em um país de média renda.

Foram definidos indicadores para monitorar e avaliar a EAAB que em sua maioria já estavam sendo utilizados. A análise permitiu atualizar os indicadores para serem mais específicos, ou seja, mais claros para capturar as mudanças desejadas em desfechos-chave, como recomendado na literatura^13^. Especialistas recomendam que os indicadores informados pela análise dos caminhos de impacto do programa sejam específicos, mensuráveis, alcançáveis, relevantes e temporais, observando se já estão em uso pelo programa ou se são planejados^13^. Um exemplo foi a atualização do indicador “número de tutores formados” para “número de tutores no município em relação ao número de tutores planejados no plano de implementação”, assim o indicador é mais específico para a adoção no nível municipal e mede a existência de um plano de formação de tutores.

Alguns indicadores da EAAB eram específicos de critérios de certificação que estavam sob revisão pelo Ministério da Saúde. Estudo prévio demonstrou que as equipes das UBS tinham dificuldade de organizar e enviar os documentos comprobatórios de determinadas atividades para a certificação e o Ministério da Saúde tinha sobrecarga de tarefas que atrasava a análise dos documentos enviados^8^. Portanto, justificou-se manter no domínio “implementação” indicadores dos critérios de certificação mensuráveis (quantitativos e com rastreamento do progresso ao longo do tempo) e alcançáveis (realisticamente mensuráveis com os dados e recursos disponíveis)^34^.

Aoki et al.^35^ utilizaram 14 indicadores baseados no *framework* RE-AIM para avaliar o estágio de implementação de um programa de promoção da saúde materno-infantil em instituições de saúde em Angola. As instituições foram classificadas em grupos de implementação ideal ou sub ótima, enquanto entrevistas permitiram complementar a avaliação e identificar barreiras e facilitadores da implementação. Em contraste, a EAAB passou primeiro por uma análise de barreiras e facilitadores para a implementação e teve documentado suas estratégias de implementação. Essas pesquisas geraram resultados positivos, especialmente na governança multinível e formação de tutores^8,12^. Entende-se que os indicadores determinados nesse estudo poderão ser futuramente utilizados para avaliar estágios de implementação e identificar municípios prioritários para monitoramento tanto da qualidade quanto da efetividade da implementação da EAAB.

Uma fortaleza do quadro de indicadores apresentado nesse estudo é que ele favorece a aplicação do *framework* RE-AIM no mundo real, concordando com especialistas da área que incentivam a ampliação do uso do RE-AIM em programas de base comunitária^31^. No Brasil, poucos estudos utilizaram o *framework* RE-AIM para avaliar programas e políticas de saúde em larga escala^36,37^. Sabe-se que o financiamento para a aplicação do RE-AIM é, muitas vezes, dependente de recursos externos dos programas, como de agências nacionais, da cooperação entre acadêmicos e serviços de saúde e de fundações^33^. O presente estudo foi conduzido como parte de pesquisa de doutorado sob financiamento de uma agência nacional de fomento à pesquisa. Assim, foi possível dedicar tempo para uma análise profunda dos caminhos de impacto do programa e dos domínios RE-AIM e propor uma estrutura de avaliação sistemática da EAAB. Portanto, salienta-se a importância da cooperação entre academia e tomadores de decisão de políticas públicas e programas complexos de saúde, para, assim, tornar factível a aplicação pragmática do RE-AIM.

Esse estudo tem limitações e fortalezas. Os dados coletados nos grupos focais são limitados às percepções dos participantes que compareceram nos encontros, e que eram apenas de municípios com contexto de implementação avançada. O segundo grupo focal teve ausência do coordenador municipal e tutor da Região Nordeste e ausência de tutores das regiões Norte e Sul. Em compensação, no processo interativo de confirmação do diagrama dos caminhos de impacto, observou-se que a maioria das sugestões do segundo grupo eram similares as do primeiro grupo, demonstrando saturação e robustez dos resultados apresentados. Alguns dos indicadores determinados para a EAAB demandam do Ministério da Saúde o envio periódico de formulários *online* para os municípios, pois eles não estão disponíveis em sistemas de informação oficiais da atenção primária à saúde. Entende-se que podem ser necessárias adaptações futuras nas fontes dos dados desses indicadores. Apesar dessa limitação, consideramos que esses indicadores devem ser mantidos como parte do sistema de avaliação da EAAB, pois se referem à existência de coordenadores, ao financiamento e ao plano de implementação, os quais são pontos críticos para controle, identificados em estudo prévio de análise da implementação da EAAB^8^.

## CONCLUSÕES

Esse estudo avançou na análise de implementação da EAAB ao determinar indicadores para avaliação tendo como referência teorias e *frameworks* da pesquisa de implementação. A teoria dos caminhos de impacto e as funções centrais confirmados funcionam como engrenagens para guiar a implementação da EAAB em larga escala de forma consistente e alcançar implementação bem-sucedida. Os conhecimentos obtidos por meio da interação com os coordenadores da EAAB das cinco macrorregiões do país apoiam a generalização dos resultados. Foram definidos indicadores para monitorar e avaliar a EAAB que, em sua maioria, já estavam sendo utilizados na implementação, entretanto, a análise com o *framework* RE-AIM permitiu atualizar os indicadores para serem mais específicos, mensuráveis e relevantes para os objetivos desejados. Em geral, os resultados do estudo apoiam a aplicação pragmática da avaliação RE-AIM em programas de saúde e incentiva o planejamento de indicadores de avaliação de outros programas de saúde e desenvolvimento infantil na atenção primária.
